# Prevalence of Intestinal Helminths among Cancer Patients Who Are under Chemotherapy at the University of Gondar Comprehensive Specialized Hospital Oncology Clinic, Northwest Ethiopia

**DOI:** 10.1155/2022/4484183

**Published:** 2022-04-18

**Authors:** Elsa Sitotaw, Adino Sitotaw, Yetemwork Aleka, Mulualem Lemma

**Affiliations:** ^1^Department of Immunology and Molecular Biology, School of Biomedical and Laboratory Science, College of Medicine and Health Science, University of Gondar, Gondar, Ethiopia; ^2^Department of Internal Medicine, School of Medicine, College of Medicine and Health Science, University of Gondar, Gondar, Ethiopia

## Abstract

**Background:**

In developing countries, environmental and personal hygiene is playing a great role in the increasing of intestinal helminth infection. In countries with limited resources and poor hygiene practices, there is a substantial overlap of intestinal helminthic and chronic infections like HIV, TB, and cancer. Intestinal helminths like *Ascaris lumbricoides*, *Trichuris trichiura*, and hookworm cause malnutrition and induce a type-2 immune response that could worsen the severity and clinical outcomes of patients with cancer. Our aim was to determine the prevalence of intestinal helminths among cancer patients who are under chemotherapy. *Methodology*. A prospective cross-sectional study was conducted in volunteer cancer patients. Clinical information were collected from study participants using a structured questioner. Stool sample was collected for parasitological examination. Formol-ether concentration technique was done, and then, two microscopic slides were prepared. Examination was done by two laboratory technicians for the detection of helminths. SPSS version 22 was used for data analysis, and simple descriptive statistical analysis was done for data presentation.

**Result:**

The total study participants were 41, of these 31 (75.6%) were females and 10 (24.4%) were male. Breast cancer and colonic cancer were the highest proportion with the others, 43.9% and 17.1%, respectively. The prevalence of intestinal parasites were 7/41 (17%). Hookworm 3/41(7.3%), *Ascaris lumbricoides* 3/41(7.3%), and *Hymenolepis nana* 1/41(2.4%) are the isolated parasite. *Conclusions and Recommendations*. The prevalence of intestinal helminths in cancer is lower than HIV and DM in the study area. However, the prevalence in these cancer patients is still high and needs deworming and health education for the better management of these cancer patients.

## 1. Introduction

Environmental and personal hygiene is very fundamental for the community as well as for the people who need special attention, since it has a great impact on the global disease burden. Low-income countries of Asia and Africa are with in high risk of environmental factors. These risk factors cause illness and death from helminth infection, respiratory infection, and diarrhea [1].

In the world, more than 1.5 billion people are infected by soil-transmitted helminths. From all these helminthes, the most common are roundworm (*Ascaris lumbricoides*), the whipworm (*Trichuris trichiura*), and hookworms (Necator americanus and Ancylostoma duodenale) [2]. Like helminths, cancer is also the major cause of mortality and morbidity in the globe, so it caused 19.3 million new cases and 10 million deaths in 2020 [3].

Intestinal helminthes have an impact on individuals by competing with micronutrients, which is very vital for the general physiology and immune response [4, 5]. On the other hand, most helminthes induce type-2 immune responses, which will downregulate the type-1 immune responses which have a pivotal role for the protection of cancer, HIV, and other infections [6].

Today, cancer is one of the big challenges of mankind, and among the 100 types of cancer, lung, stomach, liver, colon, and breast are the most fatal in each year [7]. In cancer patients under chemotherapy, their immune system is usually compromised through the disease itself and the chemotherapy they take [8]. When cancer patients under chemotherapy coinfected with helminths, their protective immune response will be highly compromised as the helminth-induced type-2 immune response could further counteract the type-1 immune response. Therefore, helminth coinfection in cancer patients will worsen the patient's condition, and that both opportunistic and nonopportunistic parasite infections become highly pathogenic. This consequence can increase the morbidity and the mortality rate of cancer patients [9, 10].

Even if certain studies have been investigating the prevalence of intestinal parasites in the community and in immune compromised patients like HIV and TB [11–13] in our country, there is no any study done on cancer patients in our setting where helminth infections are highly prevalent. Therefore, our study was aimed at evaluating the prevalence of intestinal helminthes in cancer patients under chemotherapy follow-up in University of Gondar Comprehensive Specialized Hospital, in northwest Ethiopia.

## 2. Methods and Materials

### 2.1. Study Design

Institutional based prospective cross-sectional study was conducted from February 2020 to May 2020. From cancer, these patients are under chemotherapy.

### 2.2. Study Subjects

We randomly selected 50 study participants from the outpatient unity of oncology clinic who were willing to participate in our study at University of Gondar Comprehensive Specialized Hospital. All study participants were in different stages of cancer with different cycles of chemotherapy. From these, 9 refused to give stool samples and were excluded from the study.

### 2.3. Data Collection

Independent variables and other clinical variables were collected directly from the patients and their medical record sheets by using a structured questionnaire.

### 2.4. Laboratory Methods

Stool sample was collected from the study participants and preserved by using 10% formaldehyde. Direct wet mount examination and formol-ether concentration technique were done to concentrate the helminths. Two slides from each technique were prepared for the screening of helminths by two laboratory technologists.

### 2.5. Data Entry and Analysis

All sociodemographic and clinical data and laboratory findings were entered into the SPSS version 22, and simple descriptive analysis was done.

### 2.6. Ethical Considerations

Ethical clearance was obtained from the Research and Ethics committee of School of Biomedical and Laboratory Science, College of Medicine and Health Science, University of Gondar. The participants were included after we obtained written informed consent. All personal information and identifiers of the study participants were kept conditionally, and those who were positive for any intestinal helminthes were treated with antihelminthic drugs.

## 3. Result

In our study, 31 (75.6%) participants were females, and the age groups of 29-39 and 40-50 both have 14% of the portion of the study participants. The residents of the study participants are urban 21 (51.2%) and rural 20 (48.8%) (Table 1). Breast cancer (43.9%) and colonic cancer (17.1%) were the highest proportions of the total study participants (Table 2).

From these cancer patients, their cancer stages at three were the height's proportion (20 (48.7%)) and from the total study participants. 17 (41.2%) of them had a hemoglobin level ≤12g/dl (Table 3). The overall intestinal helminth prevalence was 7/41 (17%). These helminths were hookworm, *Ascaris lumbricoides*, and *Hymenolepis nana*, which had the prevalence of 3/41(7.3%), 3/41(7.3%), and 1/41(2.4%), respectively (Table 4).

## 4. Discussion

Helminthes are the most common infectious agent of mankind and widespread in developing countries. It has been known that the morbidity of malaria, HIV, and impaired vaccine efficacy are more enhanced in Africa [14]. One of the main factors for the high burden of these morbidities in the continent is due to the high coprevalence of intestinal helminths. This might be due to the disturbance and impairment of the nutritional status of the host as a result of helminthic infections. It has been evidenced that the chronic form of intestinal helminthes with high parasitic load could reduce the absorption of vitamin A and other micronutrients (zinc, folate, and vitamin B-12) [15–17].

Moreover, helminth infection through the modulation of the immune response might contribute to the high prevalence of diseases like cancer, HIV, and other viral diseases. Most cancers and HIV need a type-1 immune response, which is a kind of inflammation by having increased inflammatory cytokines [8, 18], whereas helminth infections induce a strong type-2 immune response, anti-inflammatory. This helminth-induced type-2 immune response could suppress the protective immune response against cancer and makes the immune response not quite intact with the alteration and reduction of micronutrients in human physiology and in immune cells [19, 20].

In our study, from the included participants, the highest group of cancer patients was found in stage 3 with a proportion of 20/41(48.7%)), and there was an overall intestinal helminth prevalence of 17%. With having a poor system of early detection of cancer in the community and health facilities in third world countries, having a chronic helminth infection might have a great impact on the progression of cancer to make it worsen. This is supported by a study that showed that infection can increase the progression of cancer and the development of carcinogenesis [21].

In our study, the prevalence of *Ascaris lumbricoides* and hookworm was 7.3% for each helminth, which is lower than a study done in Brazil that found that the overall helminth infection was 66.6% and *Ascaris lumbricoides* prevalence was 33.3% [22]. In contrast, a study done in Iran showed that the total helminth prevalence in cancer patients was 6.5% and the prevalence of *Ascaris lumbricoides* was 0.5% which is lower than our results, but the prevalence of *Hymenolepis nana* was 2.7% which is similar with our study [23]. On the other hand, the prevalence of *Hymenolepis nana* in our study is higher than a study done in Egypt 0.8% [24].

A study done in Malaysian children with cancer showed that the total helminth prevalence was 42% and the *Ascaris lumbricoides* prevalence was 22% and for *Trichuris trichiura* was 24% prevalence [25]. It is higher than the findings in our study. The difference might be due to the age group of the participants in which their study is exclusively on children, which will have a more frequent contact with soil and make them highly infected by soil-transmitted helminths.

In our study area, the total prevalence of helminth parasites in cancer patients was lower than that of HIV patients (29.1% (65/223)) [26], diabetes mellitus (DM) patients (19.2% (45/234)) [27], and in community-based study (24.2%) [28]. In this study, *Ascaris lumbricoides* infection was the highest in all cancer, HIV, and diabetes mellitus patients. Ascariasis, trichuriasis, and hookworm disease are all transmitted via soil and other means that are contaminated with excreta containing infective eggs or larvae. Transmission may occur near home or in a communal area with inadequate sanitation facilities and that is polluted with faeces [29].

## 5. Conclusions and Recommendations

Even if the data has a limitation on the sample size, the prevalence of intestinal helminths in cancer patients is lower than in HIV, DM, and in the community. However, the prevalence is still high. It is better to deworm these patients in every year, and there should be a proper consultation to have good personal and environmental hygiene than the health individual, and they need special attention for early detection of infectious disease.

## Figures and Tables

**Table 1:** Sociodemographic characteristics of the study participants.

	Variables		Frequency	Percent (%)
1.	Sex	Female	31	75.6
		Male	10	24.4

2.	Age (in years)	18-28	1	2.4
		29-39	14	34.1
		40-50	14	34.1
		51-61	12	29.3

3.	Resident	Urban	21	51.2
		Rural	20	48.8

4.	Occupation	Farmer	12	29.3
		Merchant	4	9.8
		Government employed	6	14.6
		Other employment	19	46.3

**Table 2:** The distribution of different forms of cancer with respect to Hgb and BMI between the study participants.

			Stage of cancer				Hgb				BMI			
			I	II	III	IV	>13 g/dl	12-13 g/dl	10-12 g/dl	<10	<18.5	18.5-24.9	25.0-29.9	30.0-34.9
1	Breast cancer	18 (43.9%)	2	4	11	1	9	3	6	—	2	11	4	1
2	AML (acute myeloid leukemia)	1 (2.4%)	—	—	—	1	—	—	1	—	1	—	—	—
3	Bladder cancer	1 (2.4%)	—	—	—	1			—	1	—	—		
4	Cervical cancer	3 (7.3%)	—	1	1	1	1	1		1	—	2	1	1
5	Colonic cancer	7 (17.1%)	—	5	1	1	4	1	1	1	2	2	2	—
6	Chronic LL	1 (2.4%)	—	—	—	1	—	—	—	1	—	1	—	—
7	Lymphoma	2 (4.9)	—	1	1	—	1	—	—	1	—	2	—	—
8	Thyroid cancer	2 (4.9)	—		2	—	—	1	1	—	—	2	—	—
9	Hepatocellular cancer (liver)	2 (4.9)	—	1	1	—	—	—	1	1	—	2	—	—
10	Ovarian cancer	2 (4.9)	—	—	2	—	1	1	—	—	1	1	—	—
11	Lung cancer	2 (4.9)	—	1	1	—	1	—	1	—	1	1	—	—
	Total	41 (100)	2 (4.9%)	13 (31.7%)	20 (48.5%)	6 (14.6%)	17 (41.5%)	7 (17.1%)	11 (26.8%)	6 (14.6%)	7 (17.1%)	25 (61.0%)	7 (17.0%)	2 (4.9%)

**Table 3:** Clinical characteristics of study participants during stool sample collection.

	Variable		Frequency	Percent
1.	BMI (kg/m^2^)	Below 18.5	7	17.1
		18.5-24.9	25	61.0
		25.0-29.9	7	17.0
		30.0-34.9	2	4.9

2.	Hemoglobin	>13 g/dl	17	41.5
		12-13 g/dl	7	17.1
		10-12 g/dl	11	26.6
		<10 g/dl	6	14.6

3.	Stage of cancer	Stage 1	2	4.9
		Stage 2	11	26.8
		Stage 3	20	48.7
		Stage 4	8	19.5

4.	Comorbidly	HIV	1	2.4
		HBV	1	2.4
		Parasite	7	17.1

**Table 4:** Prevalence of intestinal helminths in the study participants.

	Parasite species	Frequency	Percent
1.	Hook. Worm	3	7.3%
2.	*Ascaris lumbricoides*	3	7.3%
3.	*H. nana*	1	2.4%
	Total	7/41	17%

## Data Availability

All the data that support the findings in this study found in the corresponding author upon request.

## Abbreviations

AML: Acute myeloid leukemia
BMI: Body mass index
DM: Diabetes mellitus
kg/m^2^: Kilograms per meter squared
g/dl: Gram per deciliter
HIV: Human immunodeficiency virus
HBV: Hepatitis B virus.
